# Maternal and Neonatal Outcome of Pregnant Women With SARS-CoV-2 Infection During the First and Second Wave of COVID-19 in a Tertiary Care Institute in Eastern India

**DOI:** 10.7759/cureus.22360

**Published:** 2022-02-18

**Authors:** Vinita Singh, Anisha Choudhary, Mamta R Datta, Alokananda Ray

**Affiliations:** 1 Obstetrics and Gynecology, Tata Main Hospital, Jamshedpur, IND

**Keywords:** neonatal outcome, maternal outcome, second wave, first wave, pregnancy, coronavirus, covid-19

## Abstract

Background

The ongoing coronavirus 2019 (COVID-19) pandemic is the most devastating health care crisis of our times. Pregnant women with COVID-19 infection belong to a vulnerable group with concerns about the effect of the disease on maternal and neonatal health. As we are dealing with a new disease, we must study the changing trend of disease presentation, diagnosis, and treatment to successfully manage such pregnancies.

Objective

The purpose of the present study was to evaluate the differences in presenting features, comorbidities, the fetal and maternal outcomes in COVID-19 positive pregnant women in the first and second wave of the pandemic in a tertiary care institute in eastern India.

Methodology

This study was a retrospective observational cohort study conducted at Tata Main Hospital, Jamshedpur, a tertiary care hospital in eastern India. All COVID-19 positive by reverse transcription-polymerase chain reaction or rapid antigen (RTPCR) test pregnant women (249 women) admitted to the hospital from May 2020 to August 2021 were included in this study. Out of the total, 139 women were admitted during the first wave (May 2020 to February 2021), and 110 women were admitted during the second wave (March 2021 to August 2021) of the pandemic. Data like baseline characteristics, clinical presentation, associated co-morbidities, management modalities, the maternal and neonatal outcomes were analyzed and compared.

Results

The peak of the first wave of COVID-19 was found during the months of August-October 2020, while the second wave was in April-May 2021. The majority of women had the asymptomatic or mild disease during both waves, but 14 women had moderate to severe disease during the second wave as compared to two women during the first wave. There was a significant increase in maternal deaths in the second wave (3.64%) as compared to the first wave (0.00%). During the second wave, out of 85 women who delivered, 78.8% (n=67) women had a cesarean section which was significantly higher than the first wave (64.6%). Hypertensive disorders (pre-eclampsia, gestational hypertension, and chronic hypertension) were the most common associated comorbidity, followed by diabetes (gestational diabetes, diabetes mellitus type 2) and anemia during both waves of the pandemic. The rate of preterm delivery was 27.78% (n=35) and 24.71% (n=21) during the first and second waves, respectively. Two babies tested positive within 24 hours of delivery during the first wave and one during the second wave.

Conclusion

A significantly higher number of moderate to severe disease and maternal deaths were reported during the second wave of the pandemic. A higher incidence of severe oligohydramnios and cesarean section was seen during the second wave. The frequency of preterm deliveries and low birth weight remained high during both waves. Neonatal COVID-19 infection was seen during both waves, but the incidence remained low.

## Introduction

The outbreak of coronavirus disease 2019 (COVID-19) caused by severe acute respiratory syndrome coronavirus 2 (SARS-CoV-2) has created a global health crisis. COVID-19 disease was declared as a pandemic on 11^th^ March 2020 by the World Health Organization (WHO), marking a turning point in human lives across the globe [1].

Many countries have witnessed a three-wave pattern in reported cases of coronavirus disease during the ongoing pandemic, with the first wave before August 2020 followed by the second wave in late August and September 2020 [2] and the third wave in March 2021 [3]. India reported its first case of COVID-19 in January 2020 [4] and registered a lower number of daily confirmed cases/million population in comparison to many other countries. This resulted in a prolonged first wave that lasted for over a year. The scenario started changing from March 2021 with an exponential rise in the number of positive cases and the second wave hitting the subcontinent like a tsunami. India is currently battling its third wave, with the rising number of cases being recorded each day.

With the advancement of the pandemic, our knowledge about its clinical presentations, management and complications have also evolved. Elderly, obese, and ethnic minority groups have been identified as high-risk population groups throughout the world. Evidence has shown that pregnant women also comprise a vulnerable group, with higher rates of intensive care unit (ICU) admission and mechanical ventilation when compared to non-pregnant adults of a similar age group [5]. As reported in non-pregnant adults, pre-existing comorbidities like hypertension, diabetes, high maternal age, and obesity have been identified as risk factors for severe COVID-19 disease in pregnancy [5,6].

This article aims to evaluate the differences in clinical presentation, comorbidities, the maternal and neonatal outcome in women with COVID-19 disease during the first and second wave of the pandemic in a tertiary care institute in eastern India. Mahajan et al. [7] had compared pregnancy outcomes, and COVID-19 severity among pregnant women admitted during the first and second waves of COVID-19 in a tertiary care hospital. They reported a higher frequency of severe COVID-19, ICU admission, and maternal deaths during the second wave of the pandemic. To our best of knowledge, this is the first analysis from eastern India on a comparison of maternal and neonatal outcomes during the first and second waves of this pandemic.

## Materials and methods

This is a retrospective observational cohort study of pregnant women with COVID-19 infection admitted to Tata Main Hospital, a tertiary care hospital in Jamshedpur, Jharkhand, India, from May 2020 to August 2021. All pregnant women visiting the hospital were tested as per the national testing guidelines [8] and transferred to negative, suspect, or positive isolation wards according to their COVID-19 screening status. The severity of COVID-19 was categorized as per the clinical management protocol for COVID-19 in adults [9], and women with severe disease were managed in the intensive care unit. The nasopharyngeal swab was taken from all neonates within 24 hours of delivery and tested for COVID-19 infection by RTPCR test.

Data collection

In this study, we have included 249 admitted pregnant women who tested positive for COVID-19 disease by RTPCR or rapid antigen test (RAT). Patients who refused admission or were advised of home isolation were not included in this study. The patients admitted from May 2020 to February 2021 were considered in the first wave, and those admitted from March 2021 to August 2021 were considered in the second wave of the pandemic. Retrospective data collection was done using the medical records of these pregnant women and included the baseline characteristics, past medical history, obstetric history, clinical presentation, laboratory results, management modalities, maternal and neonatal outcomes.

Statistical analysis

Statistical analysis was done with IBM Corp. Released 2013. IBM SPSS Statistics for Windows, Version 22.0. Armonk, NY: IBM Corp. Continuous variables were expressed as mean, median, and standard deviation and compared across the groups using the Mann-Whitney U test. Categorical variables were expressed as the number of patients and percentage of patients and were compared across the groups using Pearson’s Chi-Square test for independence of attributes or Fisher's Exact test as appropriate. An alpha level of 5% was taken, and a p-value less than 0.05 was considered statistically significant.

## Results

**Figure 1:**
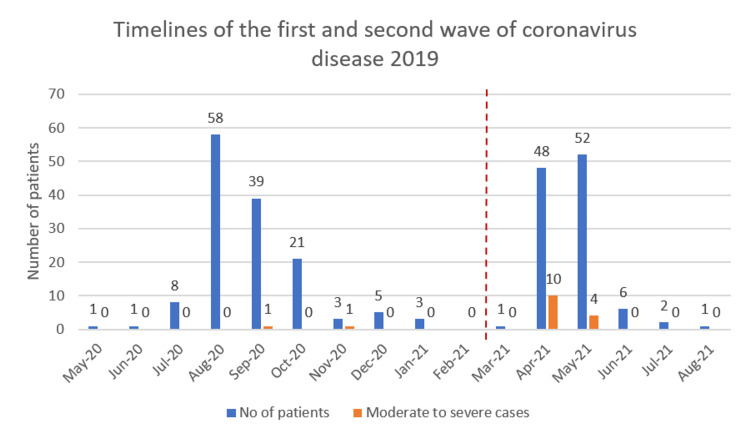
Timelines of the first and second wave of coronavirus disease 2019.

A total of 249 COVID-19 positive pregnant women were included in this study, out of which 139 women were admitted during the first wave (May 2020 to February 2021), and 110 women were admitted during the second wave (March 2021 to August 2021) of the pandemic. As shown in Figure 1, the peak of the first wave was seen from August to October 2020, followed by a steady decline and a sudden sharp rise with the second wave in April and May 2021. The number of moderate to severe cases was also significantly higher in the second wave when compared to the first wave.

The mean age of pregnant women with COVID-19 in the first wave was 27.45±4.51 years with a range of 17 years to 41 years and the mean age in the second wave was 28.45±4.45 years with a range of 19 years to 41 years. The mean period of gestation at a presentation during the first wave was 35.83±6.23 weeks and the mean period of gestation during the second wave was 33.70±8.10 weeks (Table 1).

**Table 1:** Demographic profile of the patients. ^* ^Significant at p-value <0.05

Parameter	First wave			Second wave			
	Mean	Median	Standard deviation	Mean	Median	Standard deviation	p-value
Age (years)	27.45	28.00	4.51	28.45	28.00	4.45	0.100
Period of gestation (weeks)	35.83	37.30	6.23	33.70	37.05	8.10	0.037^*^

As seen in Table 2 majority of pregnant women with COVID-19 disease had the asymptomatic or mild disease during both waves of the pandemic. However, the number of women with moderate to severe disease and requiring intensive care were significantly higher in the second wave when compared to the first wave. There were four cases of maternal deaths, all during the second wave.

**Table 2:** Severity of the disease at presentation. ^* ^Significant at p-value <0.05

Severity	First wave		Second wave		p-value
	Number	Percentage	Number	Percentage	
Asymptomatic	91	65.46	43	39.09	0.017*
Mild	46	33.09	53	48.18	
Moderate	0	0.00	10	9.09	
Severe	2	1.44	4	3.64	
Mortality	0	0.00	4	3.64	0.037*
Total	139	100	110	100	-

Hypertensive disorders (pre-eclampsia, gestational hypertension, and chronic hypertension) were the most common associated comorbidity, followed by diabetes (gestational diabetes, diabetes mellitus type 2) and anemia during both waves of the pandemic. Approximately 52% of women during the second wave had deranged liver enzymes, which were significantly more than the first wave (Table 3).

**Table 3:** Maternal comorbidities. ^* ^Significant at p-value <0.05 DM: diabetes mellitus; GDM: gestational diabetes mellitus; HTN: hypertension; GHTN: gestational hypertension

Comorbidities	First wave		Second wave		p-value
	Number	Percentage	Number	Percentage	
DM/GDM	17	12.23	16	14.55	0.593
Anemia	12	8.63	11	10.00	0.711
Cardiovascular disease	0	0.00	2	1.82	0.194
HTN/GHTN	18	12.94	16	14.55	0.612
Deranged liver enzymes	54	38.85	57	51.82	0.041^*^
Morbid obesity	2	1.44	2	1.82	0.816
Total	139	100	110	100	-

In the first wave of COVID-19, 82 women had a cesarean section, 45 women had a vaginal delivery, one patient had laparotomy for ruptured ectopic pregnancy, two patients had suction evacuation for missed miscarriage, and one patient had vaginal tear repair under anesthesia. In the second wave, 67 women had a cesarean section, 18 had a vaginal delivery, two patients had laparotomy for ruptured ectopic pregnancy, two patients had suction evacuation for missed miscarriage, and one patient had hysterotomy. A higher number of women were admitted at an earlier period of gestation and managed conservatively during the second wave when compared to the first wave (Table 4).

**Table 4:** Management after admission. ^* ^Significant at p-value <0.05

Management	First wave		Second wave		p-value
	Number	Percentage	Number	Percentage	
Conservative	8	5.76	20	18.18	0.008^*^
Suction evacuation	2	1.44	2	1.82	
Hysterotomy	0	0	1	0.91	
Laparotomy	1	0.72	2	1.82	
Caesarean section	82	58.99	67	60.91	
Vaginal delivery	45	32.37	18	16.36	
Vaginal tear repair	1	0.72	0	0	
Total	139	100	110	100	

As seen in Table 5, out of the 127 deliveries during the first wave, 64.6% (n=82) women had a cesarean section, and 45 women (35.4%) had a vaginal delivery. During the second wave, out of 85 women who delivered, 78.8% (n=67) women had a cesarean section which was significantly higher than the first wave.

**Table 5:** Mode of delivery. ^* ^Significant at p-value <0.05

Mode of delivery	First wave		Second wave		p-value
	Number	Percentage	Number	Percentage	
Caesarean section	82	64.6	67	78.8	0.032^*^
Vaginal delivery	45	35.4	18	21.2	
Total	127	100	85	100	

As seen in Table 6, women with previous cesarean admitted in labor were the most common indication for cesarean section during the first wave, and fetal distress was the most common indication for cesarean section during the second wave. This was followed by failed induction, unwilling labor trial, and malpresentation. Seven women during the second wave had cesarean section due to severe oligohydramnios, which was significantly more than the first wave.

**Table 6:** Indications for cesarean section. ^* ^Significant at p-value <0.05

Indication for cesarean section	First wave		Second wave		
	Number	Percentage	Number	Percentage	p-value
Previous cesarean	26	31.70	20	29.85	0.795
Fetal distress	22	26.80	22	32.83	0.347
Failed induction	13	15.80	8	11.94	0.287
Not willing for trial of labor	5	6.09	3	4.47	0.585
Malpresentations	5	6.09	3	4.47	0.585
Severe oligohydramnios	4	4.87	7	10.4	0.003^*^
Cephalo-pelvic disproportion	2	2.43	1	1.49	0.637
Monochorionic diamniotic twin	2	2.43	1	1.49	0.637
Nonprogress of labor	2	2.43	1	1.49	0.637
Severe preeclampsia	1	1.21	1	1.49	0.794

As shown in Table 7, during the first wave, 27.78% of women had a preterm delivery, and 24.71% of women had a preterm delivery during the second wave. Approximately 31% of total women had low birth weight babies during the second wave and 29.27% during the first wave. Four women during the first wave and two women during the second wave had intrauterine fetal death. There were three neonatal deaths during the first wave and one during the second wave of the pandemic. Two babies tested positive after birth during the first wave and one during the second wave. None of these differences was statistically significant.

**Table 7:** Neonatal outcome and complications. LBW: low birth weight; IUFD: intrauterine fetal death; NND: neonatal death; NICU: neonatal intensive care unit, COVID-19: coronavirus disease 2019

Neonatal outcome	First wave		Second wave		
	Number	Percentage	Number	Percentage	p-value
Preterm delivery	35	27.78	21	24.71	0.620
LBW	36	29.27	26	30.95	0.795
IUFD	4	2.88	2	1.85	0.699
NND	3	2.42	1	1.18	0.648
NICU admissions	26	21.31	28	33.33	0.782
COVID-19 positive	2	1.61	1	1.18	0.794

## Discussion

The ongoing COVID-19 pandemic has put an unprecedented toll on healthcare systems globally. Pregnant women belong to a vulnerable group due to concerns about the wellbeing of both the mother and the child. We had previously reported the maternal and neonatal outcomes of pregnant women with COVID-19 infection admitted from May 2020 to November 2020 [10]. In the present report, we have extended the study till August 2021 to include both the first and second waves of the pandemic, and data from both waves are compared.

During the first wave, India registered a low number of COVID-19 positive cases/million people, but the scenario unexpectedly changed in the second wave, when more than 400,000 confirmed cases/day were reported resulting in severe consequences [11]. Studies have identified various double mutant and triple mutant strains of SARS-CoV-2 across different regions of India. It is observed that the mutant virus has a more effective transmission capability with a shorter incubation period [12,13]. In our study, too, we saw a prolonged first wave with a peak during August 2020 to October 2020 and a rapidly rising second wave with a peak in April and May 2021.

The majority of the women during both the waves were asymptomatic or with mild symptoms like cough, fever, myalgia. The severity of COVID-19 was categorized as per the clinical management protocol for COVID-19 in adults [9]. There were ten women with moderate disease and four with severe disease during the second wave as compared to two women with severe disease during the first wave. There were four cases of maternal mortality due to COVID-19 pneumonia, all of whom were infected during the second wave, and none of them were vaccinated for COVID-19 infection. Mahajan et al. did a similar study among pregnant and post-partum women with COVID-19 infection in a tertiary care hospital in Mumbai metropolitan area. They reported higher rates of severe COVID-19, admission to the intensive care unit or high dependency unit, case fatality rate, and maternal mortality ratio during the second wave of the pandemic [7]. In a study by Chaudhary et al., it was reported that among the COVID-19 positive pregnant women, the need for mechanical ventilation was significantly higher during the second wave compared to the first wave. Also, the maternal death rate during the second wave was significantly high as compared to the first wave [14]. Data from the United Kingdom by Kadiwar et al. also suggested that pregnant and peripartum women experienced more severe illness in the second wave of the COVID-19 pandemic than observed in the first wave [15].

In our study, the mean period of gestation on admission during the first wave was 35.83 ± 6.23 weeks, and the mean period of gestation during the second wave was 33.70 ± 8.10 weeks. This difference was statistically significant. One possible explanation may be that majority of women were asymptomatic during the first wave and got admitted near term. During the second wave, approximately 50% of women experienced mild symptoms and were admitted in the early third trimester. Hence, a significantly higher number of women were also managed conservatively for symptomatic relief of symptoms due to COVID-19 during the second wave when compared to the first wave.

In our study, the most frequently associated comorbidities were hypertensive disorders, diabetic disorders, and anemia. There was no significant difference in the frequency of these comorbidities between the two waves. Mahajan et al. [7] reported similar findings. Approximately 51% of women during the second wave had deranged liver enzymes, which were significantly higher than that from the first wave. Several studies have reported that COVID-19 infection is associated with deranged liver function tests [16,17] and that severe acute liver injury due to COVID-19 was significantly associated with elevated inflammatory markers and a more severe clinical course, including higher rates of ICU admission, intubation, renal replacement therapy, and mortality [18]. However, these data are from non-pregnant adults, and studies on hepatic dysfunction in pregnant women with COVID-19 infection are still scarce. A higher frequency of symptomatic patients during the second wave may be a possible explanation for the significantly increased number of women with deranged liver enzymes.

The cesarean section rate was significantly higher during the second wave when compared to the first. Several studies have reported a high rate of cesarean sections during the pandemic [19-21]. This can be attributed to several factors such as maternal interest due to concern for respiratory function or a higher number of women opting for cesarean section in the isolation areas. All our patients had a cesarean section for obstetric indications only. Previous cesarean section in labor was the most common indication for cesarean section, during the first wave followed by fetal distress and failed induction. During the second wave, fetal distress followed by the previous cesarean in labor and failed induction was the most common indication for cesarean sections. We found that severe oligohydramnios was significantly more common in the second wave of the pandemic. However, only four women during the first wave and seven during the second wave had severe oligohydramnios, and this could also be an incidental finding. There are isolated case reports of severe oligohydramnios in COVID-19 infected women with low-risk pregnancy [22]; however, larger studies are required to understand the full effect of the disease on the course of pregnancy. 

In our study, we encountered a high rate of preterm births. Thirty-five (27.78%) women had preterm delivery during the first wave with mean gestational age at delivery of 34.65 weeks, and 21 (24.71%) women had preterm delivery during the second wave with mean gestational age at delivery of 33.80 weeks. In their study, Chaudhary et al. [14] saw similar findings. Many reviews report high rates of preterm deliveries among COVID-19 affected pregnant women [5,23,24], but the cause for the high preterm births remains unclear in these studies. The rate of neonatal intensive care unit (NICU) admissions was also high, being 21.31% and 33.33%, respectively, during the two waves. A living systematic review by Allotey et al. has also concluded that pregnant women infected with COVID-19 are more likely to give preterm birth and have a higher incidence of neonatal admissions to the intensive care unit [5]. The intrauterine and neonatal deaths, however, remained low during both waves. No neonatal deaths secondary to COVID-19 infection were seen in this study.

Contrary to initial reports that stated SARS-CoV-2 infection is not transmitted from the mother to the child [25], recent literature proves that vertical transmission of SARS-CoV-2 is possible and seems to occur in a minority of cases of maternal coronavirus disease in the third trimester [26]. Hosieret et al. confirmed SARS-CoV-2 invasion of the placenta, predominantly localized to syncytiotrophoblast cells at the maternal-fetal interface of the placenta [27]. In a study by Chaudhary et al. [14], two babies in the first wave and three babies in the second wave tested positive for COVID-19 infection. Similarly, in our study, two babies during the first wave and one during the second wave tested positive for SARS-CoV-2 infection. All three babies were asymptomatic and discharged with their mother.

None of the pregnant women included in our study were vaccinated for COVID-19 infection. Ministry of Health and Family Welfare, India, approved vaccination for pregnant women from July 2, 2021 [28], and we had only three COVID-19 positive women during July and August 2021, hence explaining the poor vaccination status in our study. COVID-19 infection in pregnancy can result in severe disease and rapid deterioration [5], and vaccination is an important step to protect this vulnerable group of patients. Yamini et al. recommended including COVID-19 vaccination in routine antenatal care in all countries, particularly India and Indonesia, in view of their high maternal and under-five mortality [29].

Our study had few limitations. Our data is limited to a single-center, and as our hospital is a referral center for many nearby districts, higher case rates and delays in seeking health care might have led to increased mortality during the second wave of the pandemic. Also, as we have not assessed the neonatal outcome of pregnant women infected with COVID-19 in the early trimester, the risk of teratogenicity could not be ascertained. We also acknowledge the fact that long-term follow-up is required to study the true effect of the virus on both maternal and fetal morbidity.

## Conclusions

The results of the present study show that though the duration of the first wave was longer than the second wave, a higher frequency of moderate to severe disease and maternal deaths were reported during the second wave of the pandemic. The frequency of preterm deliveries and low birth weight remained high during both waves. A higher incidence of severe oligohydramnios and cesarean section rate was seen during the second wave. Neonatal COVID-19 infection was seen during both the waves, but the incidence remained low, and all three babies were asymptomatic. The results of this study indicate that characteristics of this infection may vary over time, and future trends are difficult to predict. An alarming rise in maternal morbidity and mortality during the second wave reinforces the importance of boosting the vaccination drive for pregnant and lactating women. As we battle the ongoing third wave, it is of utmost importance that we remain vigilant and constantly study the characteristics of the disease over time so that we can modify our management protocols as deemed necessary.
